# Molecular Interactions between Saliva and Dental Composites Resins: A Way Forward

**DOI:** 10.3390/ma14102537

**Published:** 2021-05-13

**Authors:** Veaceslav Șaramet, Marina Meleșcanu-Imre, Ana Maria Cristina Țâncu, Crenguța Cristina Albu, Alexandra Ripszky-Totan, Mihaela Pantea

**Affiliations:** 1 Private Practice, 010221 Bucharest, Romania; veaceslav@saramet.eu; 2 Department of Complete Denture, Faculty of Dental Medicine, “Carol Davila” University of Medicine and Pharmacy, 010221 Bucharest, Romania; marina.imre@umfcd.ro; 3 Department of Genetics, Faculty of Dental Medicine, “Carol Davila” University of Medicine and Pharmacy, 010221 Bucharest, Romania; 4 Department of Biochemistry, Faculty of Dental Medicine, “Carol Davila” University of Medicine and Pharmacy, 010221 Bucharest, Romania; alexandra.totan@umfcd.ro; 5 Department of Fixed Prosthodontics and Occlusology, Faculty of Dental Medicine, “Carol Davila” University of Medicine and Pharmacy, 010221 Bucharest, Romania; mihaela.pantea@umfcd.ro

**Keywords:** dental caries, dentin, saliva, composite resins

## Abstract

Dentin and enamel loss related to trauma or especially caries is one of the most common pathological issues in dentistry that requires restoration of the teeth by using materials with appropriate properties. The composite resins represent dental materials with significant importance in today’s dentistry, presenting important qualities, including their mechanical behavior and excellent aesthetics. This paper focuses on the saliva interactions with these materials and on their biocompatibility, which is continuously improved in the new generations of resin-based composites. Starting from the elements involved on the molecular landscape of the dental caries process, the paper presents certain strategies for obtaining more advanced new dental composite resins, as follows: suppression of oral biofilm acids formation, promotion of remineralization process, counteraction of the proteolytic attack, and avoidance of cytotoxic effects; the relation between dental composite resins and salivary oxidative stress biomarkers is also presented in this context.

## 1. Introduction

Dental composite resins represent some of the most used materials in modern dentistry. Owing to the growing and successful interaction of interdisciplinary fields of materials science and molecular biology, demand for biomedical materials has increased in recent years. With such dynamicity, the dental materials have advanced a lot [1].

The primary interest of material scientists and dental medicine researchers is the development of new dental materials characterized by reduced polymerization shrinkage, better healing, and mechanical and aesthetic properties [1]. Composite resins represent dental materials providing a very good biocompatibility within the oral environment [1,2]. Biocompatibility represents the ability of a biomaterial to sustain a specific function in the context of a medical treatment without generating any harmful local or systemic effects, but inducing the most advantageous cellular or tissue responses, in a given situation, optimizing and completing in this way the medical therapy [3].

Current research studies are focused mainly on correlating composite resins physical and chemical properties with their oral biocompatibility. Certain aspects are of great interest in the study of dental composite resin behavior in the oral environment, as follows: The compounds released by these resins during and after polymerization process; the degradation of the bonding agent [4]; their cytotoxic effects on gingival, pulpal cells, and oral stem cells [5]. Stem cells, due to their remarkable ability to differentiate into specific cell lines, are more and more regarded as a new hope in repairing and regeneration of dental pulp lesions. Therefore, a future, true challenge will be represented by the molecular interactions between the oral stem cells and dental composites [5].

The improvement of composite resins formula with compounds that could provide antiseptic properties [2] represents an important topic in the medical scientific research. Currently, nanotechnology and nanoscience also play an important role in development of new dental composite resins [6].

Various dental materials, such as glass ionomer cements, silicates, or resin-based composites, have now been produced for a long time. Composite resins are considered highly appreciated dental restorative and root filling materials, due to their special esthetic properties and their ability to counter compressive forces within oral cavity [7].

The aim of this paper is to synthesize the complex interactions of dental composite resins with the oral environment, especially with saliva: suppression of biofilm acids formation, remineralization process, counteraction of the proteolytic attack, avoidance of cytotoxic effects, oxidative stress related issues. This approach reflects the valuable properties of dental composite resins, as well as certain aspects regarding adverse effects potentially caused by them. The elements addressed in this paper have, also, a practical connotation, as dentists should be permanently informed and aware about the biomechanical properties of dental materials—especially of the new ones—and should personalize the selection of restoration materials, in accordance with the complexity of each clinical case.

## 2. New Elements from the Chemical Landscape of Novel Composite Resins

Taking into account the above-mentioned aspects, the main strategies for elaborating efficient new composite resins, are: (1) suppressing biofilm acids production; (2) promoting remineralization; (3) counteracting the proteolytic attack; and, importantly, (4) avoidance of the cytotoxic effects.

### 2.1. Suppression of Biofilm Acids Production

Acidogenic bacteria, able to ferment carbohydrates, are the principal actors on the cariogenic process initiation stage [8]. The secondary carious lesion represents a main reason for restoration failures [9]. Consequently, it is highly recommended to obtain new materials in order to inhibit acid producing bacteria growth. Knowing that restorative materials are able to interact with oral biofilms, it is important to develop new materials with therapeutic properties. These properties should include: (1) inhibition of biofilm adhesion, (2) inhibition of biofilm growth, (3) biofilm metabolism alteration, and (4) balancing the microbial ecosystem [10].

Composite resins are increasingly used in dentistry due to their esthetic properties and direct filling capabilities [11]. Unfortunately, these materials encourage the accumulation of biofilms and acids producing bacterial plaque, triggering recurrent caries formation at margins [12,13].

Recently, there have been developed new antibacterial monomers: dimethylaminododecyl methacrylate (DMADDM) and dimethylaminohexadecyl methacrylate (DMAHDM) [12,13]. Both these monomer types have enhanced antibacterial activity compared to quaternary ammonium dimethacrylate (QADM). These monomers’ long polycationic chains are able to interact with the bacterial membrane. It has been proven that the carbon chain length plays an important role in the antimicrobial activity [12,13]. For instance, DMADDM, with a longer carbon chain of 12 atoms, revealed stronger antimicrobial activity compared to DMAHM, with a shorter chain of only 6 carbon atoms [12,13]. Moreover, silver nanoparticles and DMADDM incorporation into an adhesive system, has significantly reduced the biofilm metabolism, without affecting the dentin bond strength [12,13].

DMADDM and amorphous calcium phosphate (NACP) nanoparticles integration into adhesive systems and composite resins triggered a more efficient antimicrobial activity, reduced pulpal inflammation, and intensified the reparative dentin formation [12,13]. It, also, has been highlighted that the 10% DMAHDM bonding agent can completely destroy the S. mutans biofilm in vitro [12,13]. Composites containing the combination of DMAHDM with NACP reduce colony-forming units (CFU) of S. mutans, significantly decrease biofilm metabolic activity and, consequently, the acid production [12,13].

Chlorhexidine (CHX) and silver (Ag) particles have been used as releasing agents in various studies concerning composite resins properties improvement [11,12,13,14]. The bacterial cell membrane in contact with CHX is rapidly damaged. However, this damage is unable to induce apoptosis or cytolysis [11,12]. CHX is also able to traverse by passive diffusion the bacterial cell membrane, and to induce cytosolic lesions, leading to apoptosis [11,12]. Other studies outlined the special antimicrobial activity of the silver ions, which can be used effectively to prevent infections [15,16]. We should mention here the strategy of loading this Ag particles into the filler of the composite, rather than to the resin matrix [13].

Silver (Ag) particles have also been incorporated in resins [17,18]. Chatzistavrou et al. results revealed that Ag containing resins had efficient antimicrobial activity against S. mutans and Escherichia coli [19]. Morones et al. suggested that Ag ions are able to inactivate vital bacterial enzymes leading to the loose of replication ability and cell death [20].

One of the important limitations of these releasing systems is the uncontrollable burst release, which can last only for a short time [21,22]. This inconvenience can be overcome using covalently bonded (“immobilized” and “non-releasing”) agents [21,22]. Immobilization of non-releasing antimicrobial agents in resins can be achieved by copolymerizing quaternary ammonium methacrylates (QAM) in the resin matrix [21,22]. 12-Methacryloyloxydodecylpyridinium bromide (MDPB) copolymerization in a resin matrix induced an efficient and durable contact inhibition of bacteria cells [21]. Similar results were reported using quaternary ammonium polyethyleneimine nanoparticles [23,24], methacryloxyethyl cetyl dimethyl ammonium chloride-containing adhesive [25], antimicrobial glass ionomer cements [26], antimicrobial composites [27], and antimicrobial nanocomposite and adhesive [28].

The quaternary ammonium salts antimicrobial mechanism is illustrated by their binding capacity to cell membranes, finally causing cytoplasmic leakage [21,23]. When the QAM resin’s positively charged (N^+^) atoms contact the negatively charged bacterial cell surface, the cell membrane electrochemical balance may be altered, possibly leading to the cell lysis, under osmotic pressure [21,23]. Quite recently, a series of QAMs with different alkyl chain lengths (3, 6, 12, 16, and 18 carbon atoms) have been synthesized [28]. Experimental data revealed that the short-chained quaternary ammonium’s antimicrobial activity rely probably on the positively charged ammonium groups which can interact with the negatively charged bacterial membrane surface. These interactions will lead to the alteration of the biological functions of the membrane, essential ions (Na^+^, K^+^, Ca^2+^, and Mg^2+^) balance disruption, inhibition of different enzymes activity and bacterial DNA damage [29]. It has also been reported that the increase in chain length led to reduced bacterial metabolism, and, consequently, a decrease in organic acid production and release [30]. DMAHDM with an alkyl chain with 16 carbon atoms showed the strongest antimicrobial activity [30]. The QAM molecules with long alkyl chains have two killing effects:

1 One based on the positively charged ammonium groups
2 The second, relying on the long alkyl chain, able to insert into the bacterial membrane, triggering structural and metabolic damages in the bacterial cell [28,29].

However, one of the limitations of QAM-containing resins is that the salivary proteins tend to cover the resin surface. This resulted protein coat on the resin’s surface will prevent the QAM resin surface–bacteria contact, consequently reducing the efficacy of the “contact killing” process of QAM [21]. Considering this disadvantage, it is of great interest the development of protein-repellent resins. These dental materials would have two important advantages:

1 Preventing the salivary protein coat formation on the resin’s surface, the QAM sites will be exposed to bacterial cells, in order to initiate and proceed contact-killing;
2 Regarding the bacterial plaque formation, protein adsorption on the resin surface represents a mandatory condition for bacterial attach [31].

Muller et al. reported that immobilized poly-(ethylene glycol) and methacrylate monomers with pyridinium groups produced protein-repellent functions [32]. Lewis et al. used 2-methacryloyloxyethyl phosphorylcholine (MPC). MPC is a methacrylate with a phospholipid polar group [33]. The protein-repellent molecular mechanism relays on the fact that, usually, the hydrophilic surfaces usually tend to prevent protein adsorption, compared with hydrophobic surfaces [34,35].

Phospholipids represent major components of cell membranes [36]. Phospholipid molecules consist of hydrophobic tails and hydrophilic heads. In water, these molecules will organize themselves into a bilayer in which the nonpolar tails are oriented in the inner area, while the polar heads are oriented outwards and interact electrostatically with the water dipoles. MPC polymers are hydrophilic and by hydration they become surrounded by many free water molecules [35]. Ishihara et al. considered that these free water molecules around the phosphorylcholine groups are ensures salivary protein molecules repelling efficiently [35]. Zhang et al. have shown that the MPC copolymerized dental composite proved to be an efficient protein repellent [37]. Moreover, similar results were obtained with both MPC and DMAHDM copolymerized resin [37].

### 2.2. Promoting of the Remineralization Process

In order to protect teeth against secondary caries, an important step would be to produce composite resins containing reactive fillers with anticariogenic properties. These dental materials are known as “bioactive restoratives” [38]. A new class of biocomposites called alkasites (including Cention N) are now available. These composite resins contain a mixture of glasses, especially an alkaline calcium fluorosilicate glass. Within oral environment, mainly if it has an acidic pH, alkaline calcium fluorosilicate glass composites are able to releases ionic species, among which the fluoride ions will play a main role in the remineralization process.

Recently, Tiskava et al. highlighted that on Cention N surface apatite formation is possible, ensuring in this way the underlying dentin remineralization [39]. Cention N liquid component consists of a four non-acidic monomers combination and does not have any adhesive properties. The special properties of this new dental material are based on its powder component, which contains incorporated reactive fillers [40]. The manufacturer has recommended Cention N for all temporary tooth restorations, for posterior teeth occlusal and proximal restorations and, also, for cervical restorations in combination with an adhesive. Due to its presence on the market for quite short time, there are very few research studies that have analyzed all its biological advantages [41,42].

The use of antibacterial bonding agents is very important in order to eliminate residual bacteria from the tooth cavity and overwhelm new invading bacteria at the edges, without altering the bond strength [21]. In this regard, Wang et al. studies on *S. mutans* have revealed that DMADDM containing adhesives were able to trigger bacterial cells’ growth limitation, acid production, and exopolysaccharides synthesis inhibition, without affecting the dentin bond strength [43].

Quaternary amine has also been investigated as a possible candidate for bonding agents, using 3-dimensional biofilms of S. mutans [44]. The results of Zhou et al. illustrate that the charge density should be regarded as a key element in antibacterial resins development because the positive charges interact with the negatively charged bacteria cell surface, triggering an efficient antimicrobial activity [44]. The studies of Gerdes et al. and Murata et al. highlighted that materials with high density cationic surfaces create a stress condition able to trigger apoptosis initiation in bacterial cells [45,46].

Nevertheless, more scientific studies are needed in order to investigate in detail these resin-biofilm interactions and their effects on the surrounding oral tissues.

### 2.3. The Counteracting of the Proteolytic Attack

During the cariogenic process, in the early stage of demineralization, salivary and dentinal proteolytic enzymes (like MMPs and cathepsins) may induce serious alterations of the carious dentin matrix. These proteolytically induced alterations can interfere with the mineralized tissue repairing process, although demineralization has been chemically stopped [47]. This is the reason why dentinal and salivary MMPs and cathepsins should be regarded as key actors on the cariogenic process stage. These enzymes should become important targets for the therapeutic strategies of carious lesions. So, now it is the MMPs inhibitors’ turn to enter the scene.

Resin polymers chemical and structural stability is considered to be essential for the adhesive-dentin interface longevity. The degradation of either, adhesive molecules or dentin, may weaken the adhesion and cause gaps formation between tooth surface and the restorative material [48].

In vitro and in vivo research studies highlighted that the hybrid layer (HL) generated by the dentin bonding systems are vulnerable in aqueous medium, because both resins [47,48,49] and collagen fibrils [50,51,52,53] may suffer hydrolytic degradation processes. Given that MMPs are regarded as important players in the dentin matrix breakdown during caries lesions progression, it is logical to consider these proteolytic enzymes responsible for the collagen fibrils disintegration, within the HL [47,53,54].

The resin impregnation of dentinal cavities is quite frequently incomplete, as a result, the denuded collagen fibrils, associated with free water, are exposed to enzymatical cleavage, triggering the HL degradation [48,49,52,53,54]. In vitro and in vivo research studies revealed that the use of nonspecific enzyme-inhibiting systems ensured, to some extent, the increase in HL longevity [48,49,52,53,54]. Galardin, chlorhexidine, and benzalkonium chloride have been used as therapeutic primers in the bonding system and provided positive results [48,49,52,53,54]. Remineralization is a very important chemical process for the positive evolution of a carious lesion [55]. The composite resin remineralization activity is provided by calcium phosphate particles incorporation into the resin matrix [51].

The amorphous calcium phosphate (ACP) composite releases calcium and phosphate ions. These ionic species may sustain the remineralization of carious lesions [55]. Unfortunately, the ACP particles composites do not have mechanical resistance and cannot be considered bulk restoratives [55]. However, Xu et al. have managed to improve the composite mechanical properties, and at the same time to ensure the release of high levels of calcium and phosphate ions, by incorporating NACP into the resin macromolecule [56]. Moreover, Chen et al. have included the NACP into an adhesive system with 5% DMADDM [56]. Chen results revealed that the adhesive was able to release phosphate and calcium ions to support remineralization and had very efficient antimicrobial action, illustrated by the inhibition of biofilm acid production [56]. Much better effects have been obtained by Melo et al. by including 3 agents into the composite resin macromolecule: the remineralization agent, NACP, and two antibacterial agents, Ag and DMAHDM [57].

It is certain that further research studies are still necessary in the composite resin development field, however the technique of multiple bioactive agents’ incorporation into resins macromolecules should represent an important target and a promising starting point.

### 2.4. Avoiding the Cytotoxic Effects

Dental composite resins are widely used as restorative materials in dental medicine due to their important advantages, such as: easy handling possibilities, controlled working time, and very good aesthetics [58]. The evaluation of biocompatibility and cytotoxicity of any dental material is as important as the assessment of its physical properties [59].

However, one of the problems related to composite resins use in dentistry is represented by the monomers. If the composite resin polymerization reaction was inadequate, unpolymerized monomers may remain in the resin matrix. These unreacted monomers can be released in the oral cavity [59]. Such monomers have been identified in the saliva immediately after the composite resin restorations application [60].

Moreover, there is the probability that some organic chemical species from resin macromolecules may be released in small quantities, as a result of the exposure to an oxidative micro-medium and to salivary or bacterial enzymes activities. In this regard, there have been reported interesting data regarding the cytotoxic effects of different composite resins. Chaharom E et al. and Kamlak et al. concluded that different composite resins’ cytotoxicity depends primarily on their chemical composition, synthesis method, and structural characteristics [61,62].

Despite the continuous improving of the resin’s composition, this type of dental materials is still synthesized by polymerization of methacrylate monomers via vinyl bonds [61,62]. The widespread use of this synthesis method is based on the many advantages of the methacrylate’s polymerization:

(1) The possibility to obtain at room temperature conversion degree values of >60% to 70% in less than 1 min, using on-command photo-activating methods
(2) Methacrylate’s polymerization generates resistant macromolecular networks, at room temperature
(3) The resulted polymers have adequate mechanical properties
(4) These polymers can be used for highly esthetic restorations [61,62].

Despite these important advantages, a main problem is that methacrylates are susceptible to hydrolysis reactions, due to their ester bonds. These hydrolysis reactions may be stimulated by pH decrease and/or temperature increase [63], conditions that can occur in the oral cavity.

Besides, the incomplete conversion may cause the release of soluble unreacted monomers into saliva and, last but not least, reduces the polymer matrix stability, triggering a shorter clinical lifetime [63].

Experimental data revealed that methacrylamide monomers were more stable in aqueous medium, even in acidic conditions [64,65]. Methacrylamide monomers have been proposed to be used as:

1. adhesives monofunctional monomers; and
2. multifunctional monomers for composites [65].

Bis-acrylamides proved to be very suitable for the crosslinkers roles in dental composite resins. The resulting macromolecule revealed an improved stability in aqueous medium and physical properties comparable to those of methacrylates polymers [64]. Gonzalez-Bonet et al. reported that the vinyl ethers proved to be very stable, even in the presence of hydrolytic enzymes [66]. However, the homopolymerization of these monomers, presented above, is not possible at room temperature. Consequently, it is necessary a heat treatment after photoactivation or they should be copolymerized with methacrylate monomers [66].

The copper-catalyzed azide-alkyne click polymerization reactions are very efficient and generate macromolecular networks with high mechanical resistance at room temperature [67].

The materials obtained by thiol-vinyl sulfone polymerizations have networks with conversion degree of almost 80%, due to efficient photo-base generator initiators development [68]. Besides, in these materials, the monomers’ molecular structure can be adapted in order to avoid degradable esters formation, and to obtain macromolecular networks highly resistant to solvents action [68].

The work by Nishida et al. conducted on odontoblast-like mouse cells, illustrated that 12-Methacryloyloxydodecylpyridinium bromide (MDPB) had reduced cytotoxic effects compared to bisphenol A glycidyl methacrylate (BisGMA), leading to the conclusion that MDPB had better biocompatibility than BisGMA [69].

Li et al. in vitro studies revealed that DMADDM also produced less cytotoxic effects than BisGMA [70].

Imazato et al. in vivo studies, conducted on dog teeth, revealed that the experimental MDPB containing primer induced no pulpal inflammation, compared with the moderate inflammation reaction, generated in MDPB absence, in the control group. The adhesives with antimicrobial properties have been recommended for pulp care, given that MDPB incorporation in these materials did not affect the original material’s biocompatibility [27].

Imazato’ s results are consistent with the Li et al. study conducted on composite resins and adhesive materials containing NACP and DMADDM. NACP and DMADDM composite resin use triggered moderate pulpal inflammation and initiated more efficiently the tertiary dentin generation, versus controls [70].

All these results highlight that the bioactive composite resin and adhesive materials with incorporated NACP and DMADDM have a remarkable biocompatibility, are able to sustain dentin-pulp complex healing processes, and, last but not least, have the capacity to reduce the biofilm acid production by inhibiting oral pathogen bacteria growth.

### 2.5. Composite Resins and Oxidative Stress—A Possible Cause and Effect Relationship

The in vitro study of Pantea et al. revealed that the tested composite resins did not induce significant changes of the salivary redox status markers (uric acid, gamma glutamyl transferase, and oxidative stress responsive kinase-1) [2]. The tested dental composite resins were: a resin for direct dental restorations—GC G-ænial Posterior/GC Corporation/Tokyo/Japan—that have an organic matrix (UDMA/Urethane dimethacrylate/dimethacrylate co-monomer), filler, pigments and photo-initiators; a resin for indirect dental restorations—Gradia Lab Indirect Restoration System/GCCorporation/Tokyo/Japan—a nano-hybrid composite, having ultra-fine fillers with very high density and homogeneous distribution, mixed in the resin matrix; a composite resin for cementation of indirect dental restorations—G-Cem Link Force//GCCorporation/Tokyo/Japan—an universal dual cure resin cement.

The obtained results of this study (that pointed out that the tested resins do not interfere with salivary oxidative stress), together with other reported data concerning composite resins’ biocompatibility, have clinical applicability, and highlight, increasingly, the special biological properties of these modern dental materials [2].

In practical terms, informed selection of dental resin composites, in correspondence to each specific clinical case, and their use in accordance with the producers’ recommendations are of great importance, as well as long term monitoring and well scheduled periodical follow-ups of the patients in order to signal possible adverse reactions or post-treatment side effects.

Furthermore, as long as the development directions of medical scientific research include individualized treatment strategies, personalized and precision dentistry, it is possible that, in the future, we will witness at the design of drug combination specific to each individual, personalized drug delivery, continuously modulated treatments, depending on each individuals’ health state. Additionally, it is estimated that personalized dental restorative biomaterials will be improved, based on the important acquisition of data at the individual level (biomarkers), including at the salivary level [71].

In this context, this paper provides relevant data with practical applicability, and further stimulates the interest in developing scientific research on the interaction between saliva and dental composite resins.

## 3. Conclusions

Currently, the scientific research in the composite resins’ field is oriented, increasingly, towards the biofilm–host interactions. This paper highlights certain important strategies in obtaining new dental composite resins, with improved ability to integrate within the special environment which characterize the oral cavity. Analyzing the research activity in the dental polymer chemistry field, it results that the organic phase of composite materials represents a crucial subject. A suitable molecular structure of the organic monomers which undergo polymerization reactions, represents the first step on the way towards a complex dental material with high biocompatibility, antimicrobial capacity, healing and remineralizing abilities, adequate aesthetic and good mechanical properties.
